# Meaning above (and in) the head: Combinatorial visual morphology from comics and emoji

**DOI:** 10.3758/s13421-022-01294-2

**Published:** 2022-03-02

**Authors:** Neil Cohn, Tom Foulsham

**Affiliations:** 1 Department of Communication and Cognition, Tilburg School of Humanities and Digital Sciences, Tilburg Center for Cognition and Communication, Tilburg University, P.O. Box 90153, 5000 Tilburg, LE Netherlands; 2 Department of Psychology, University of Essex, Colchester, UK

**Keywords:** Visual language, Morphology, Affixation, Compositionality

## Abstract

Compositionality is a primary feature of language, but graphics can also create combinatorial meaning, like with items above faces (e.g., lightbulbs to mean inspiration). We posit that these “upfixes” (i.e., upwards affixes) involve a productive schema enabling both stored and novel face–upfix dyads. In two experiments, participants viewed either conventional (e.g., lightbulb) or unconventional (e.g., clover-leaves) upfixes with faces which either matched (e.g., lightbulb/smile) or mismatched (e.g., lightbulb/frown). In Experiment 1, matching dyads sponsored higher comprehensibility ratings and faster response times, modulated by conventionality. In Experiment 2, event-related brain potentials (ERPs) revealed conventional upfixes, regardless of matching, evoked larger N250s, indicating perceptual expertise, but mismatching and unconventional dyads elicited larger semantic processing costs (N400) than conventional-matching dyads. Yet mismatches evoked a late negativity, suggesting congruent novel dyads remained construable compared with violations. These results support that combinatorial graphics involve a constrained productive schema, similar to the lexicon of language.

## General introduction

The compositionality of language has often been taken as a hallmark of linguistic structure, yet abstract, combinatorial meaning-making also appears in other modalities, like visuals. For example, hearts may float above a head or substitute for eyes to show lust, or a lightbulb above a head may indicate inspiration. While this type of “visual morphology” is stereotypical of comics (Cohn, 2013, 2018; Forceville, 2011, 2016; McCloud, 1993), it has proliferated across emoji and filters for photographs, where such morphemes can be applied to human bodies. These combinations require a comprehender to link modifying information (like hearts, lightbulbs) to a more stable form (like a characters’ head) to derive a meaning beyond their parts. Because of these relationships, these combinations have been compared with affixes in the morphology of language (Cohn, 2013, 2018; Engelhardt, 2002; Forceville, 2011), making them “lexical items” in “visual languages” of graphics which may vary across cultures (Cohn, 2013; Cohn & Ehly, 2016; Forceville et al., 2010; Tasić & Stamenković, 2018). Because these forms integrate different sources of meaning in noniconic ways, and are growing in ubiquity throughout society, they provide a way to investigate combinatorial meaning-making outside the domain of spoken or signed languages.

### Theories of visual affixation

Visual Language Theory (Cohn, 2013) posits that drawn information uses structural and cognitive principles similar to that of language. This is particularly salient in the combinatorial properties of visual morphology. In contrast to visual forms that can easily stand alone, some visual representations must attach to other forms. A speech balloon must connect to a speaker, while a motion line depicting a path must attach to a moving object. These forms cannot retain their meaning when free floating unconnected to another visual element. Because of this dependent nature, these forms have been likened to *bound morphemes* in language, which must affix to a more primary *stem* (Cohn, 2013, 2018; Engelhardt, 2002; Forceville, 2011). Thus, a speech balloon is a visual affix which attaches to the stem of a speaker. This dependent nature may also be hierarchical. Consider the lightbulb that floats above characters’ heads to convey inspiration. Typically, the lightbulb also has radial lines that emanate from it to depict its brightness. Here, the radial lines affix to the lightbulb, and this composite then affixes to the stem of a person. Thus, visual affixes appear to attach in a hierarchical structure (Cohn, 2018).

In addition, like in linguistic systems, visual morphology uses various physical strategies to combine bound morphemes with their stems (Cohn, 2013, 2018; Forceville, 2019). Affixes can physically attach to stems through juxtaposition, such as lightbulbs above the head, speech balloons, or motion lines. They can also substitute for parts of a stem, as in linguistic suppletion, such as replacing eyes with hearts, or dotted lines replacing solid lines to show invisibility. Finally, parts of an image may be repeated, similar to reduplication in language, such as repeating a body part multiple times to show that it is moving. These basic strategies of attachment, substitution, and repetition reflect the variety of possibilities for forms to connect with each other, and thereby are abstractly similar across verbal and graphic modalities.

Visual morphemes may also vary in the way they convey meaning. Some have stable meanings (the heart shape, speech balloons, motion lines) while some in context change from iconic to symbolic meaning (lightbulbs for inspiration, gears for thinking), possibly invoking conceptual metaphors that draw on the iconicity to convey such symbolicity (Cohn, 2018; Forceville, 2011, 2016; Szawerna, 2017). In such cases, an emergent, construed meaning arises that is greater than the sum of the parts: Nothing about a happy face or a lightbulb alone convey inspiration, but together they create this emergent meaning (Cohn, 2018). These conventionalized meanings are recognized easily by most viewers (Cohn et al., 2016). It should be noted that this again aligns with linguistic morphology, such as the construal of meaning between words within compounds, which runs the gamut of conceptual relationships (Jackendoff, 2009).

While Visual Language Theory hypothesizes a relationship between the structure and cognition of language and visual representations, such relations exist at an abstract level. For example, it is not that “affixation” in visual representation “is like” or “is parallel to” that of linguistic morphology, but rather that basic strategies of cognition operate in both the verbal and visual forms in similar ways. Whether both modalities recruit overlapping domain-general mechanisms is an empirical question. This issue is explored for visual morphology in the current research (Experiment 2) but is also suggested by prior work showing similar neural responses to the processing of visual narrative sequences as to sentences (Cohn, 2020b), and that verbal information can be modulated by a visual sequential context, and vice versa (Federmeier & Kutas, 2001; Ganis et al., 1996; Manfredi et al., 2017; Weissman & Tanner, 2018).

### Visual affixation and memory

Given that visual affixation involves the construction of meaning out of disparate parts, it begs the question of how this information is encoded and processed. Theories of processing visual morphology also take on the character of linguistic theories (Leminen et al., 2019). One possibility is that combinatorial visual morphemes are stored in memory on an item-by-item basis. This type of *full-entry theory* of lexical storage as applied to words would imply that derivations of different words—both regular and irregular—are independently stored as a whole in the lexicon (Jackendoff & Audring, 2020). Applied to visual morphemes, individual affixes would then become encoded in long-term memory individually, with a “lexicalized” meaning that is unique and conventionalized (Kennedy, 1982; McCloud, 1993; Walker, 1980). Evidence for such encoding comes from findings that the frequency of conventionalized morphology, and familiarity with them, influences their comprehension (Cohn et al., 2016; Nakazawa, 2016; Newton, 1985).

**Fig. 1:**
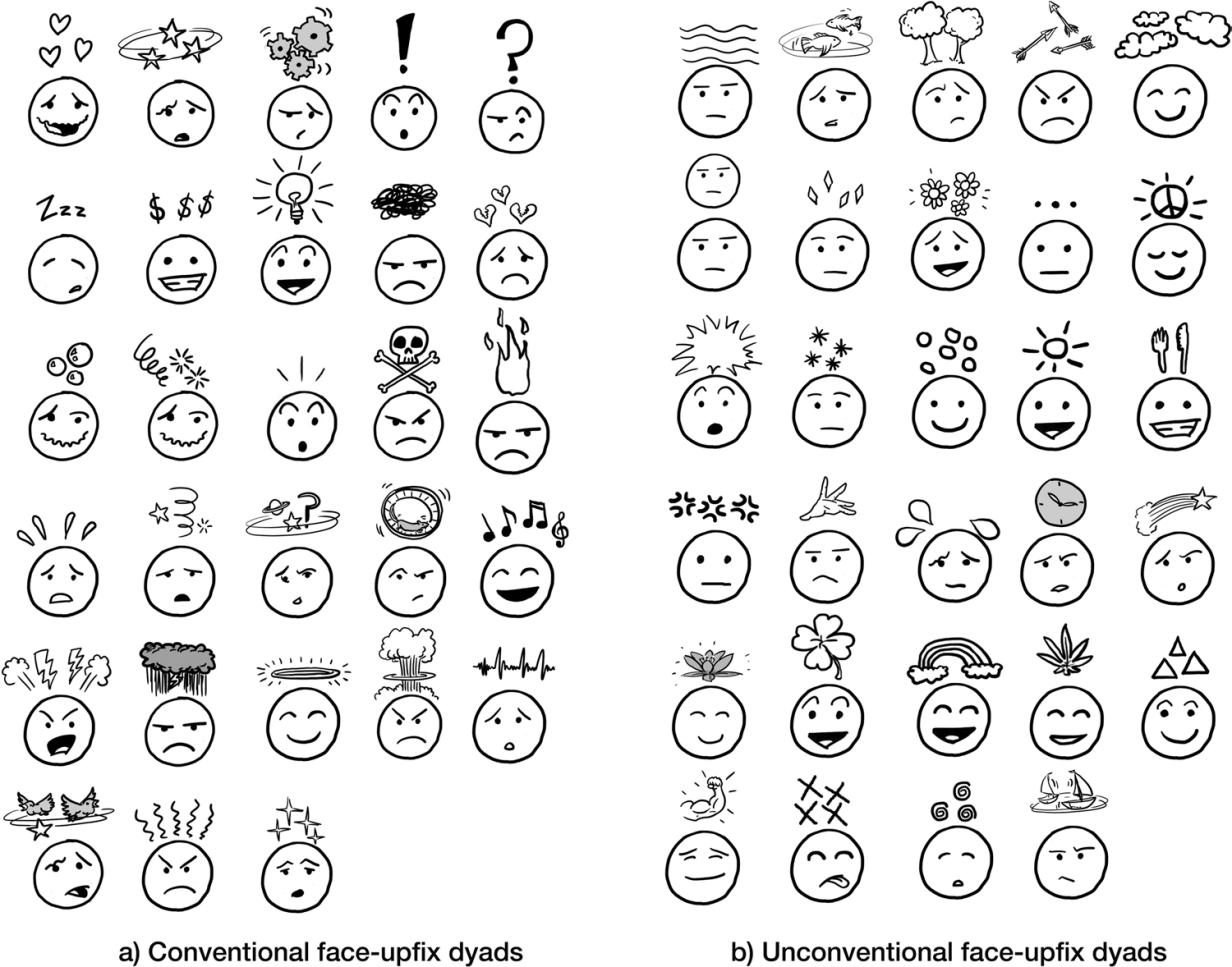
Visual morphology of “upfixes”—elements placed “up” above a face to result in a meaning related to a cognitive or emotional state. **a** Depicts conventional upfixes frequently found in visual media (comics, cartoons, emoji), while **b** depicts unconventional upfixes which are novel, produced for this study

A full-entry theory would posit that understanding of visual morphemes involves the retrieval of specific stored meanings from memory, with little interaction from the stems themselves (Feng & O’Halloran, 2012; Ojha, 2013). In other words, placing squiggly lines above a head should evoke the meaning of anger (Fig. 1a), regardless of the facial expression of the stem. Thus, novel affixes, like those in Fig. 1b, would be construable as an independent meaning if they used conventionalized representations (such as peace signs), but others might be viewed as incongruous or meaningless as upfixes until they become encoded in memory.

An alternative perspective views that, though visual lexical items may be entrenched in memory, their compositional meaning is dynamically construed given their context (Bateman & Wildfeuer, 2014). Such a *dynamic-construal theory* renders visual morphology equal across different degrees of conventionality, and across their combinatorial interaction with other elements (like faces). Here, whether they are conventionalized or not, meanings of visual morphemes would be computed online with reference to their discourse context. Indeed, context allows many visual morphemes to be understood beyond their item-based encoding. Some affixes change in meaning based on their position (Cohn, 2013, 2018; Forceville, 2011; McCloud, 1993), such as stars above the head to mean dizziness, stars replacing eyes to mean a desire for fame, and stars adjacent to a body part to mean pain. Similarly, three “spiking” lines might mean surprise (third row in Fig. 1a), but next to a gold bar would mean shininess. Across these cases, the same visual representations change meaning depending on their context, and a dynamic-construal theory would thus extend this notion to all visual morpheme contexts.

Nevertheless, variation by position could remain conventionalized by item, and indeed the variable interpretations of stars (above head vs. in eyes) rely on conventionalized meanings for those different positions. If dynamic construal alone guides comprehension, it implies that *all* combinations are somehow construable, and none are truly incongruous. Such an extreme view has not been supported by experimentation where participants both explicitly and implicitly recognize some visual morphological combinations as more felicitous and meaningful than others (Cohn et al., 2016; Cohn & Maher, 2015; Ojha, 2013). Nevertheless, emergent meanings do arise from unconventional combinations between visual affixes and stems, implying that some construal process is at work (Cohn et al., 2016).

Within Visual Language Theory, we have proposed that some forms of visual morphology can become abstracted into productive classes (Cohn, 2013, 2018). In this *lexical-schema theory*, some visual elements may be lexicalized as instances, while belonging to a generalized template of a “lexical schema” that allows for novel combinations. This conception of a visual lexicon is consistent with constructional theories of morphology and the lexicon in language (Booij, 2010; Jackendoff & Audring, 2020), where productive lexical items are stored as declarative schemas with variables that are filled by specific instances. This architecture allows for lexical items (e.g., words) to be stored as memorized instances (aware*ness*, happi*ness*) but also to allow for abstraction of novel forms (comic-*ness*, emoji-*ness*) across the generalized structure (X-*ness*).

For bound visual morphemes, this schematic nature is exemplified by elements that float above characters’ heads, such as lightbulbs, hearts, gears, circling stars, and many others, as in Fig. 1a. These forms are argued to comprise a class of visual affixes placed above characters’ heads, and because these affixes are “up” from their stem, we call them *upfixes* (Cohn, 2013, 2018). These cases thus are posited to involve a schema with open variables for the upfix above a character’s head, and also a variable for the facial expression of the character. This overall relationship facilitates construal of upfixes to typically have meanings related to the cognitive or emotional state of the character.

This schema is further hypothesized to specify constraints on the position and relationship of an upfix to its stem. First, upfixes are constrained to a location above the head, and may appear incongruous if moved too far from an upward position. Second, the facial expression of the stem must “agree” with the upfix: A storm cloud upfix should appear awkward above a happy face, while a lightbulb should seem awkward above a confused face (Cohn, 2013). This combination allows upfixes and faces to work together in creating a singular construal, clarifying facial emotions that may be ambiguous on their own (Stamenković et al., 2018). Because upfixes are argued to involve a productive lexical schema, novel forms floating above characters’ heads could “fill the slots” of the schema that remain interpretable as some form of cognitive or emotional state. As such, novel forms should also adhere to these constraints of position and face–upfix agreement.

**Fig. 2:**
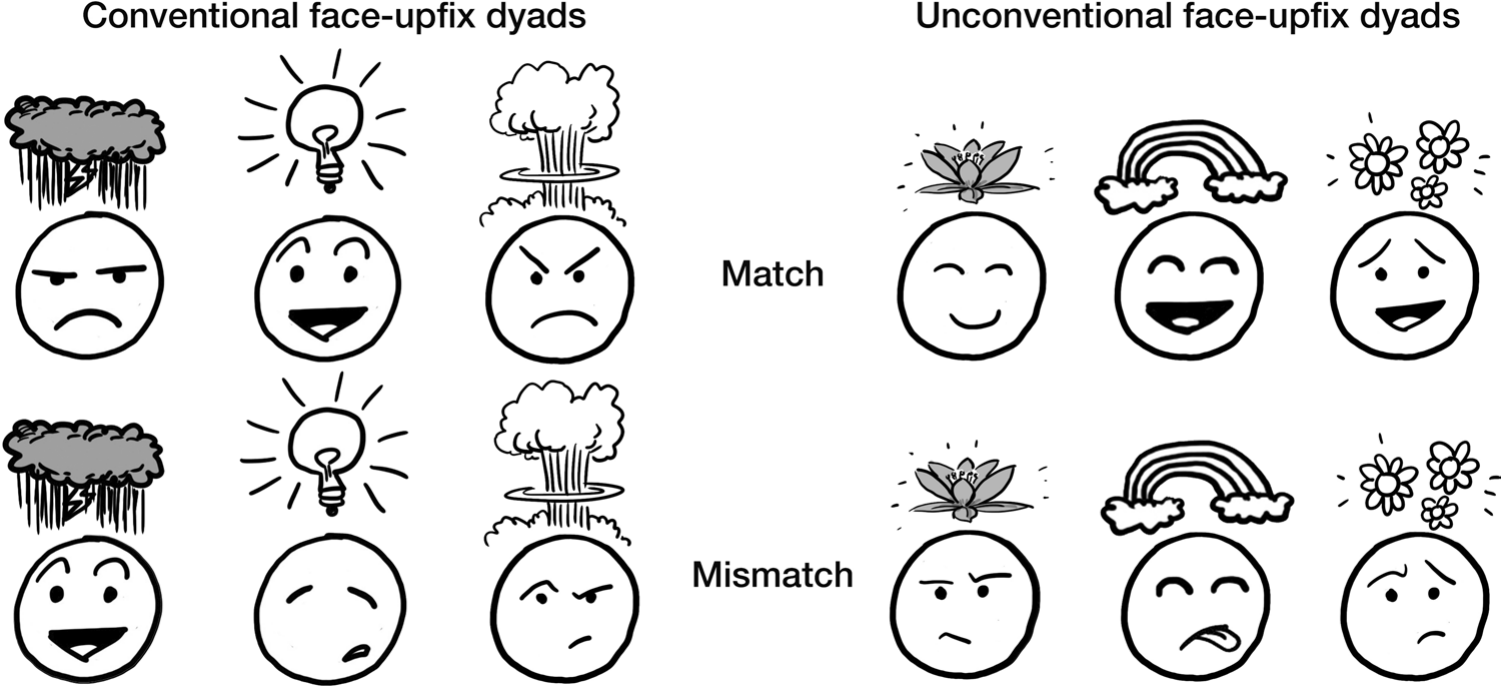
Conventional and unconventional upfixes where the upfix either matches or mismatches the facial expression

In prior research (Cohn et al., 2016), we examined the idea that upfixes formed a productive class governed by constraints on the relationship between the face and upfix (whether they matched or mismatched, as in Fig. 2) and their relative positions (above or beside the head). Upfixes that were beside the head were rated as less comprehensible than those above the head, while those mismatching the facial expression were rated even lower. These constraints also interacted, as mismatching faces that were also beside the head received the lowest comprehensibility ratings of all. These results suggested that upfixes are indeed constrained by both the spatial location relative to a face, and its facial expression. However, these same constraints appeared to operate on both conventional and unconventional upfixes. Though novel, unconventional upfixes were rated as less comprehensible than conventional ones, spatial location and facial mismatch again modulated these assessments.

This provided evidence that upfixes use a productive schema, and not simply full-entry memorized items, because these constraints operated on both established upfixes and novel ones. It also provided evidence against a full dynamic-construal theory (Bateman & Wildfeuer, 2014), since unconventional and/or mismatching dyads were less comprehensible than conventional ones. Moreover, participants overtly commented on the lack of ability to combine mismatches into a coherent, holistic interpretation, going against the idea that they would be dynamically construed.

Subsequent research examined response times and eye-movements to matching and mismatching face–upfix dyads that compared whether the face and/or upfix was in a cartoony or photorealistic style (Kendall et al., 2020). Here, participants’ response times did not differ based on face–upfix matching for recognizing the overall emotion of the dyad, which was also not modulated by the style of the face or upfix (cartoony or photorealistic). Participants fixated on faces prior to upfixes but spent more time dwelling and fixating on upfixes than faces as long as the face was cartoony. Analyses using more graded emotional valence of dyads found longer response times when the emotions of face–upfix dyads were more ambiguous than when they were more clearly matching or mismatching (Kendall, 2019).

While this research provided initial evidence for the schematic nature of upfixes as a productive class of visual morphology, the behavioral evidence for the matching constraint (response times) was mixed and focused on conventional dyads. Stronger evidence for an upfix schema would come from contrasting how these constraints operate across the conventionality of dyads. We therefore conducted two studies examining the constraints of conventionality and face–upfix matching to better assess the combinatorial structure of these visual morphemes. In both experiments we presented participants with the component parts of the dyad (face, upfix) before the composite face–upfix dyad, measuring the response times for participants’ judgements of their comprehensibility (Experiment 1) and their electrophysiological brain responses using event-related potentials (Experiment 2). This work thus aimed to further assess the processing of face–upfix relationships.

## Experiment 1: Viewing times

Only a few studies have yet analyzed face and upfix relationships with behavioral measures. Prior work found that response times to recognize the congruency of face–upfix dyads did not differ between matching and mismatching dyads (Kendall et al., 2020), and these times did not differ depending on whether the face and/or upfix was in a cartoony or photorealistic style. Kendall (2019) further examined dyads with incremental changes in emotion: faces shifted from happy to neutral to sad expressions, while upfixes shifted from a sun to sun-with-clouds, and then only rain clouds. These graded upfixes were paired with faces so that they were gradually matching (happy-sun to sad-rain) and mismatching (happy-rain to sad-sun). Participants recognized the congruency of dyads along this gradient, but their response times to these judgements were slowest to medial mismatching (e.g., semi-happy face with semi-stormy upfixes) compared with relatively faster times to fully matching and mismatching dyads. These findings were thus mixed for how mismatching affects response times, but do not address how they operate in fully unconventional dyads.

In order to address these concerns, our first study presented participants with face–upfix dyads where the visual morphological elements (face/upfix) were presented one at a time prior to the compositional whole. We manipulate the (mis)match between face and affix across both conventional and unconventional dyads, as in Fig. 2. These stimuli were presented using a self-paced viewing task where participants had to assess how well they understood the meaning of the images at each screen, and we measured their response times to these judgments of comprehensibility.

If upfixes were stored in memory on an item-by-item basis, conventional upfixes should be better than unconventional ones, with no advantage of matches over mismatches. Conversely, if comprehension is entirely based on construals, no differences should appear across our types: conventional matching face–upfix dyads would receive no advantage to unconventional or mismatching dyads, because they would all involve the same process of construal. These extreme positions are unlikely though, as our prior studies have shown sensitivities to conventionality and mismatching. If processing follows prior judgements (Cohn et al., 2016), to provide evidence of a productive schema, we expect that coherent conventional upfixes would be easier to process than unconventional ones, which should both be easier to process than mismatches, which violate the structure.

### Methods

#### Stimuli

We used the stimuli from our previous study of upfixes (Cohn et al., 2016) consisting of 58 face–upfix dyads, including 28 conventional dyads (Fig. 1a) and 30 unconventional ones (Fig. 1b). Conventionality was confirmed using ratings measured from our prior study (Cohn et al., 2016: Experiment 1) for how familiar participants were with these dyads along a 1 (*not familiar*) to 7 (*very familiar*) scale. Conventional dyads had an average score of 5.8 (*SD* = .86), with unconventional dyads scoring an average of 3.4 (*SD* = 1.1). For each of these dyads where the face and upfix “matched,” we created mismatching relationships where the facial expression mismatched the upfixes, as in Fig. 2. Mismatches were again confirmed using ratings from our prior study (Cohn et al., 2016). This resulted in 116 total face–upfix dyads crossing conventionality and (mis)matching.

An additional 38 faces with non-upfix morphology were used as fillers to increase the heterogeneity of the stimuli and to prevent participants from anticipating the above-the-head positioning of the affix prior to the dyad. These included both conventional and unconventional face–affix pairs that were substituted for eyes (e.g., hearts or stars for eyes), on the forehead (vertical lines for dread), out the nose (bubble for sleep), or other idiosyncratic signs (like a zipper for a mouth). Variations also used signs that were similar to those used as upfixes but were then placed in a different position. This ensured that viewers would not know how faces and affixes/upfixes would be arranged. Thus, if a person saw a question mark and face in the first two images, they would not know until the final image if it would become an upfix (conventional) or would have the question mark come out of the nose (unconventional). These non-upfix morphemes were also presented in matching and mismatching types to amount to 76 total non-upfix arrangements.

To control for the order of information viewed by a participant, we presented stimuli either as face first, then upfix, then the full dyad, or upfix first, then face, then together. In total, this amounted to 232 upfix stimulus types across conventionality, matching, and order, and 152 non-upfix morphemes. Here, we collapse across these orders and analyze stimuli only at the face–upfix dyads.

Because of the large numbers of items throughout our whole stimulus set, we divided these stimuli into lists such that each participant saw 58 total dyads across our stimulus types. This comprised 29 upfix dyads (seven or eight of each of the four upfix types) and 29 non-upfix dyads (nine or 10 matching or mismatching). While this meant that each participant did not see all morphemes, across all participants, all dyads across all types were viewed in all presentation orders.

#### Participants

Our participants were 82 individuals (31 females, 51 males, mean age = 34.6 years, *SD* = 11.2) recruited online through social media. All participants gave their informed consent to participate. Pretest questionnaires assessed participants’ familiarity with reading and drawing various types of comics using the Visual Language Fluency Index (VLFI). VLFI scores have previously been shown to correlate with behavioral and neurocognitive aspects of visual narrative processing, including ratings of upfixes (e.g., Cohn, 2020a; Cohn et al., 2016). Participants’ VLFI scores covered a wide range (1.5–52.5), but on average they were very proficient comic readers, with a mean of 22.4 (*SD* = 12.3), where below 7 is low and above 20 is high fluency.

#### Procedure

Participants accessed the experiment through an online survey website (Qualtrics), where they first filled out the VLFI forms and then proceeded to the primary experiment. The self-paced viewing task used the jspsych plugin (de Leeuw, 2015). Each trial began with a screen reading READY. When pressing a button, participants progressed through each of the three screens of the trial (face–upfix dyad or upfix–face dyad). At each screen in the trial, they were instructed to make a yes/no forced choice for whether they understood the meaning of the dyad. Because all trials were fixed at three screens each, this incremental judgement task was done so that participants would not speed through the stages without paying attention. At the end of the experiment, participants were asked for any further comments they may have and were thanked for their participation.

#### Data analysis

Our analysis focused on participants’ response times and comprehensibility judgments at the combined face–upfix dyad. For each participant, we averaged response times and comprehensibility judgements across individual items in a subjects analysis. For response times, we removed outliers that were below 200 ms or above 2.5 times the standard deviation of the mean. We used a 2 × 2 ANOVA with within-subjects factors of Conventionality (conventional, unconventional) and Matching (match, mismatch) to analyze both our response times and comprehensibility judgements. Post hoc pairwise analyses used a Bonferroni correction.

A follow-up items-analysis of response times examined whether conventionality affected response times in a more graded way across dyads. We used the ratings of each face–upfix dyad’s conventionality (1 = *not familiar*, 7 = *very familiar*) from our prior study (Cohn et al., 2016: Experiment 1). Averaging across response times for each participant, we here correlated response times for both matching and mismatching dyads with the average conventionality scores for each dyad from Cohn et al. (2016).

### Results

#### Comprehensibility judgments

**Fig. 3:**
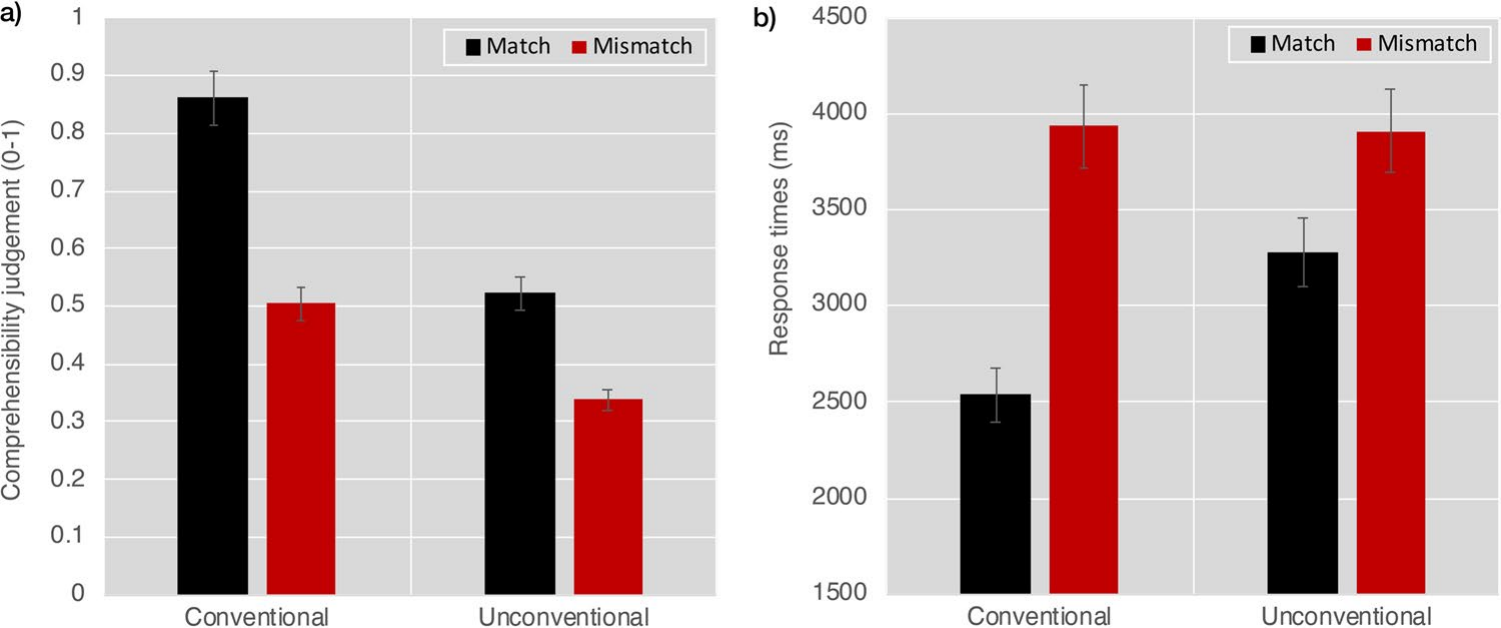
Results of **a** mean comprehensibility ratings and **b** response times in milliseconds to assessments of face–upfix dyads that varied on dimensions of conventionality and the matching of the facial expression to the upfix. Error bars depict standard error

We first analyzed participants’ assessment of the comprehensibility of the face–upfix dyads which revealed a main effect of Conventionality, *F*(1, 81) = 166.2, *p* < .001, a main effect of Matching, *F*(1, 81) = 148.8, *p* < .001, and an interaction between them, *F*(1, 81) = 34.4, *p* < .001. These results arose because participants were more likely to rate conventional matches as comprehensible, less likely to rate unconventional mismatches as comprehensible, and with ratings of unconventional matches and conventional mismatches in-between, as depicted in Fig. 3a. All contrasts between dyads were significantly different (all *p*s < .001), except between the intermediate ratings of unconventional matches and conventional mismatches.

#### Response times

We next analyzed how long participants took to make their comprehensibility judgements. We found a main effect of Conventionality, *F*(1, 81) = 9.2, *p* < .005, a main effect of Matching, *F*(1, 81) = 48.2, *p* < .001, and an interaction between them, *F*(1, 81) = 12.4, *p* < .001. As depicted in Fig. 3b, these results arose because mismatching face–upfix dyads were responded to slower than matching dyads (all *p*s < .005), and while mismatching dyads did not differ in their response times based on conventionality (*p* = 1), participants responded to conventional matching dyads faster than to unconventional matching dyads (*p* < .001).

**Fig. 4:**
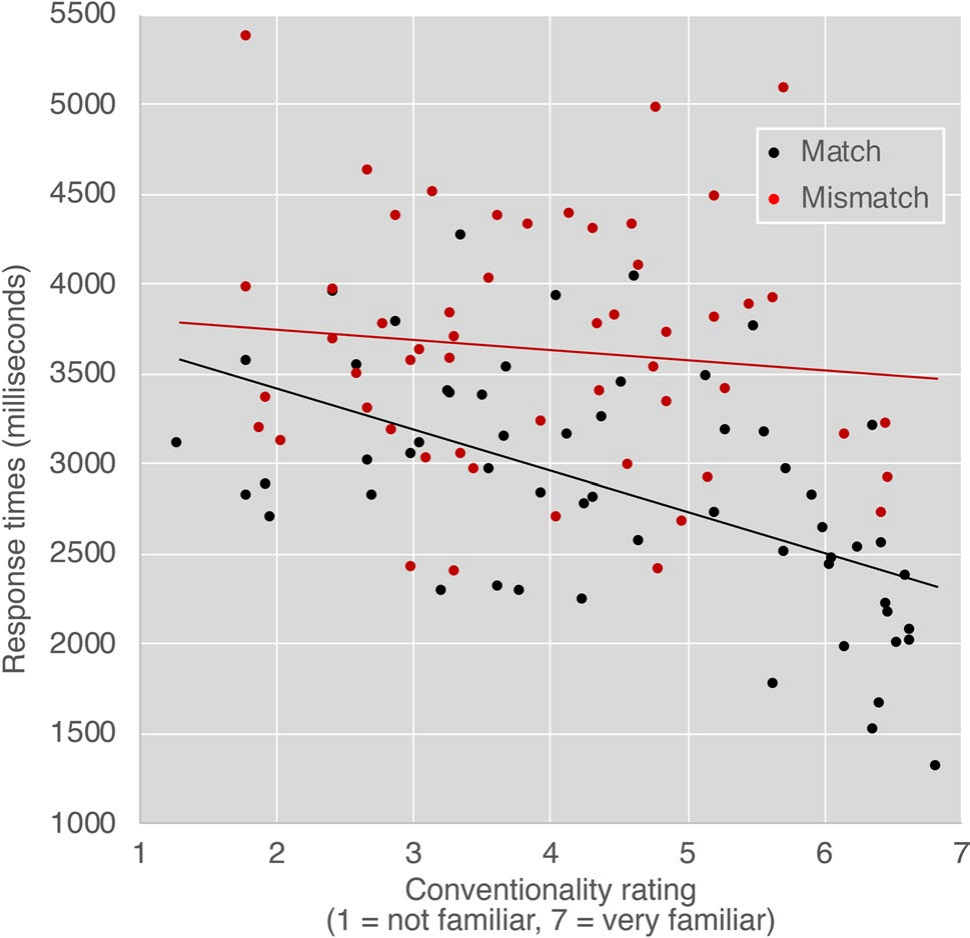
Correlations between conventionality ratings from Cohn et al. (2016) and response times to ratings of comprehensibility for matching (black) and mismatching (red) face–upfix dyads. Each datapoint indicates a different dyad

We next asked whether conventionality persisted in a more graded way. We correlated the ratings of conventionality for each dyad gathered in a previous study (Cohn et al., 2016) with participants’ response times for both matching and mismatching dyads. As depicted in Fig. 4, a negative correlation suggested that greater conventionality for matching dyads led to shorter responses times, *r*(55) = −.549, *p* < .001. However, the correlation between conventionality scores and mismatching dyads was not significant, *r*(55) = −.107, *p* = .443.

We found no correlations between VLFI scores with either ratings or response times.

### Discussion

This study examined the compositionality of, and constraints on, productive and nonproductive visual morphology. Consistent with prior findings (Cohn et al., 2016), conventional dyads were rated as more comprehensible than unconventional dyads, but within both types, mismatching face–upfix dyads were rated as less comprehensible than matching dyads. Thus, because even novel, unconventional dyads evoke certain constraints on face–upfix agreement, it suggests that these constraints operate across an abstract schema.

Participants’ response times to making these judgements supported these results but displayed different relationships between the dyad types. Here, mismatching face–upfix dyads were responded to slower than matching dyads, yet mismatching dyads did not differ across conventional and unconventional types. This suggested that the mismatching relationship between faces and upfixes drove the increase in response times, beyond conventionality. These results differ from prior work that did not evoke varying response times between face–upfix dyads on the basis of (mis)matching (Kendall, 2019; Kendall et al., 2020). Those prior studies used a smaller range of upfixes and emotional states as stimuli, with an additional contrast between cartoony and photorealistic images, which may have mitigated the congruency effects. Because mismatching incurs a processing cost similarly to both unconventional and conventional upfixes, it implies that upfixes involve a productivity that can be violated regardless of their familiarity.

Though conventionality did not affect response times across mismatching dyads, it did modulate those of matching face–upfix dyads. Here, conventional dyads were responded to faster than unconventional dyads, which were both responded to faster than mismatches. These faster times to more conventional dyads occurred in a graded way, but again only for matching face–upfix dyads. Here, we showed that faster response times occurred on an item-by-item basis based on their conventionality scores (Cohn et al., 2016). This is consistent with previous findings that familiarity with face–upfix dyads increases their comprehension (Cohn et al., 2016; Newton, 1985). The faster times to conventional dyads further support that they have an advantage in processing over both mismatches and unconventional matching relationships. This implies that conventional matching dyads—the standard types—are stored in memory as part of a visual lexicon (Cohn, 2013; Forceville, 2011; Walker, 1980), while their differences with unconventional dyads implies that they are not construed uniformly in the absence of entrenched representations (cf. Bateman & Wildfeuer, 2014).

In sum, the higher ratings and faster response times to conventional dyads compared with unconventional dyads supports some degree of encoding of item-based types in memory. However, because mismatches are rated lower than matches for unconventional face–upfix dyads, and because mismatching unconventional dyads incur a similar processing cost in response times as mismatching conventional dyads, it supports the presence of a schematic template abstracted beyond encoded types.

## Experiment 2: Event-related potentials

In Experiment 1, conventionality modulated response times to matching face–upfix dyads but did not influence mismatching dyads. However, response times do not allow us to assess whether the cognitive processes at work during meaning-making are similar across conventionality and (mis)matching relationships. In Experiment 2, we thus measured event-related brain potentials (ERPs) to the same manipulations as in Experiment 1 in order to better assess the neurocognitive processing involved in these interactions. ERPs are a measure of the electrophysiology of the human brain in online processing, time-locked to particular stimulus events (here, the onset of each image).

Given that participants consciously rate unconventional and mismatching face–upfix dyads as less comprehensible (Cohn et al., 2016), we might expect such manipulations to create costs for how they create meaning. Indeed, prior work (Cohn et al., 2016) showed that the conventional face–upfix dyads evoke more stable interpretations for their meaning than mismatches or unconventional dyads. In ERPs, semantic processing is indexed by the “N400,” a negative polarity deflection peaking around 400 ms (Kutas & Federmeier, 2011). Though first discovered in the context of language (Kutas & Hillyard, 1980) and modulated by morphology (Leminen et al., 2019), the N400 has been well attested as an index of semantic processing across domains, including visual scenes, pictures, and visual narratives (Barrett & Rugg, 1990; McPherson & Holcomb, 1999; Sitnikova et al., 2008; Võ & Wolfe, 2013; West & Holcomb, 2002). The N400 is thought to index the access or retrieval of information in semantic memory, regardless of the modality (Kutas & Federmeier, 2011).

Because the N400 is sensitive to incongruities, we would expect that mismatching, and perhaps also unconventional, face–upfix relationships will evoke larger N400s than conventional matching dyads, because only the conventional dyads are encoded in memory. Mismatching faces with upfixes might be likened to irregular or violated morphology of words, which has been observed to elicit larger N400s than regular morphology (for review, see Leminen et al., 2019). For the dimension of conventionality, the N400 has long been shown to be sensitive to frequency effects across modalities. In language, high lexical frequency leads to attenuated N400s compared with low frequency or novel lexical items (Barber et al., 2004; Kretzschmar et al., 2015; Kutas, 1993; Leminen et al., 2019; Van Petten, 1993, 2014), while familiarity or repetition of pictures also attenuates the N400 (Curran & Cleary, 2003; Schendan & Kutas, 2002). Given these findings, similar frequency effects could be observed for the conventionality of upfixes, leading to attenuated N400s for the conventional matching upfixes.

Infrequency might also factor into the N400 for mismatching face–upfix relationships. If the N400 indexes a process of integration or construal beyond the retrieval of memory-based representations, the N400s to mismatches should be greater than to matching unconventional dyads. However, such integrative processes have often been observed to effects subsequent to the N400. Sustained negativities occur for persisting interpretive or inferential processing in both sentence and discourse contexts (Baggio, 2018; Bott, 2010) and in visual narratives (Cohn, 2020b), and strong, sustained negativities have been observed to novel compound words (Fiorentino et al., 2014). If novel upfixes demand an additional construal process than entrenched conventional upfixes, we may thus expect sustained negativities associated with that process.

In addition, later positivities have been associated with the updating or revision of a mental model of a representation (Baggio, 2018; Cohn, 2020b; Donchin & Coles, 1988; Kuperberg, 2016), and such processes could be posited as occurring to the integrative relationship between a face and upfix when they mismatch. Indeed, late positivities were evoked in a study of motion lines in comics—the lines that trail a moving object. When the lines reversed to depict a backwards action/motion in a panel from a visual narrative sequence, they evoked both larger posterior and anterior positivities than regular motion lines (Cohn & Maher, 2015). Reversed motion lines violate the structural expectations of their schematic relationship, and thus we might predict similar positivities to the violations introduced by our face–upfix mismatches. Indeed, similar positivities have been found to structural violations of naturalistic scenes, such as objects being placed in unexpected locations (Võ & Wolfe, 2013).

We also considered the possibility that upfixes might evoke differential ERPs associated with face processing. In a comparison of visual styles of emotional faces (Kendall et al., 2016), cartoony faces evoked an attenuated P1 when compared with photorealistic and rotoscoped faces. This was attributed to the low-level featural differences between cartoony and photorealistic faces, which were posited as feeding forward into the subsequent N170—an ERP component reflecting the neural processing of faces (Bentin et al., 1996) which is also sensitive to emotional expressions (Blau et al., 2007) and is modulated in comparisons of real faces and emoji faces (Gantiva et al., 2020; Weiß et al., 2020).

Subsequent research then compared iconic cartoony faces with versions where a novel symbol was replaced for the mouth, a “suppletion” similar to the more conventional zipper for a mouth (Kendall, 2019). In a first presentation, these symbolic representations evoked a larger and earlier latency N170 compared with the relatively meaningless symbolic-mouth faces. However, participants then underwent a training phase that taught them to recognize these mouth-symbols as indicative of different emotions. Subsequent measurements of ERPs showed that the faces with mouth-symbols then evoked a greater N170 than the iconic faces, which was also greater than the symbolic-mouth faces in the pretraining measurement, though they remained at a later latency. Such results suggest that with a conventionalized understanding, this combinatorial visual morphology becomes processed similarly to iconic faces.

Interestingly, an apparent later deflection in the waveforms of this study remained unanalyzed. Here, the learned symbolic-mouth faces appeared to evoke a greater negativity (i.e., greater negative amplitude effect) than those from before the learning phase, yet both remained less negative than the iconic faces, which did not differ across phases. This negativity occurred between 200 and 300 ms with a peak around 250 ms, consistent with an N250, a posterolateral waveform implicated in both memory and perceptual processes which has been shown to be greater to faces that are more familiar than those that are less familiar (Begleiter et al., 1995; Schweinberger et al., 1995), whether that familiarity was encoded in memory or newly acquired (Tanaka et al., 2006). While primarily shown to faces, it has also been extended to familiarity with a complex visual representation (Scott et al., 2006). Such results have suggested that the N250 may index “perceptual expertise” for the access or formation of stored perceptual representations (Folstein et al., 2017; Schendan & Ganis, 2015). The implication for the Kendall (2019) study is that the reduced N250 arose to these symbolic-mouth faces because they were not familiar to participants, though the training phase enhanced their conventionality leading to a greater N250 (Folstein et al., 2017; Scott et al., 2006).

These results suggest that unconventional visual morphology (like our unconventional upfixes) can evoke early effects associated with visual complexity and familiarity. Nevertheless, these studies by Kendall presented stimuli with a fairly short duration of 300 ms, making it difficult to examine downstream components such as the N400. Here, we therefore aim to further examine the neurocognition of combinatorial visual morphology. In line with the research reviewed above, we predicted that, like Kendall (2019), we would observe a reduced N250 to unconventional relative to conventional dyads. However, we expected that both conventionality and mismatching would lead to larger N400s, as they would require more costs to the access and/or integration of meaning. If both mismatching and conventionality separately contribute to such access, we might expect a graded or additive effect, as in the results of Experiment 1. Finally, unconventionality and mismatching may also influence later effects (late positivities, late negativities), although without prior findings we did not have specific expectations.

### Methods

#### Stimuli

The same stimuli were used in Experiment 2 as in Experiment 1 (Table 1). However, rather than divide stimuli into different lists, all participants were presented with all stimuli in all conditions in a within-subjects design. This meant that each condition was viewed roughly 60 times (4 dyad types × 2 orders). Trial order was randomized uniquely for each participant, presented using PsychoPy2 (Peirce et al., 2019).

**Table 1:** Total items used in the experiments

	Upfix First Order	Face First Order	Total
Conventional Match	28	28	56
Conventional Mismatch	28	28	56
Unconventional Match	30	30	60
Unconventional Mismatch	30	30	60
Total upfixes	116	116	232
Matching non-upfix fillers	38	38	76
Mismatching non-upfix fillers	38	38	76
Total fillers	76	76	152

In Experiment 1, items were distributed into 8 counterbalanced lists, each with 48 items (29 upfixes, 29 non-upfix fillers), while in Experiment 2, all items were shown to all participants

#### Participants

We recruited 20 participants from Tilburg University (seven males, 13 females; mean age = 23.1 years, range: 19–28) who were compensated with course credit for their participation. All participants gave their informed written consent to take part in the study. Participants were all dominantly right-handed with normal or corrected-to-normal vision, no history of traumatic head injury, and taking no psychoactive medications. The VLFI range was wide, from very low (2.63) to a high average (20) with an overall mean of 9.37 (*SD* = 4.49). However, participants all rated themselves as frequent users of emoji both currently (*M* = 6.15, *SD* = 1.55) and while growing up (*M* = 4.25, *SD* = 2.1) on a 1 (*never use*) to 7 (*always use*).

#### Procedure

Participants sat in a comfortable chair across from a computer screen in a soundproof chamber. At the start of each trial, participants saw a screen reading “Ready” where they were instructed to press to begin. A red dot in the center of the screen gave them a fixation point which persisted throughout the experimental trials. After pressing to begin a trial, images appeared in succession one at a time in the center of the screen. Images remained on screen for a duration of 750 ms with a 300 ms ISI, after which a question mark appeared on the screen. Participants then rated the final image (face–upfix combination) for how easy it was to understand on a scale of 1 (*hard to understand*) to 7 (*easy to understand*). When the whole experiment was finished, participants filled out an open-response posttest questionnaire asking them to describe anything “unusual” or any patterns they may have noticed throughout the experiment.

#### Data analysis

EEG was measured using a Brain Products ActiChamp and 32 channel Standard actiCAPs with the sampling rate set to 250 Hz and a high cutoff filter of 70 Hz. Electrode Fz was used as a reference electrode, and electrodes beside the left eye and beneath the right eye monitored for eye movements and blinks. We kept electrode impedances below 10 kΩ for all electrodes. We used the ERPLAB plugin for EEGLAB in MATLAB (Lopez-Calderon & Luck, 2014) to refilter the raw EEG data offline with a bandpass filter of .1-30 Hz, and then we re-referenced the data to the average of the mastoid channels (TP9, TP10). Trials were epoched from 0 to 800ms with a prestimulus baseline of 200 ms. Trials with excessive blinking or muscle artifact were removed from analyses, which lead to excluding the data of one participant from our final analysis (>10% rejected trials).

**Fig. 5:**
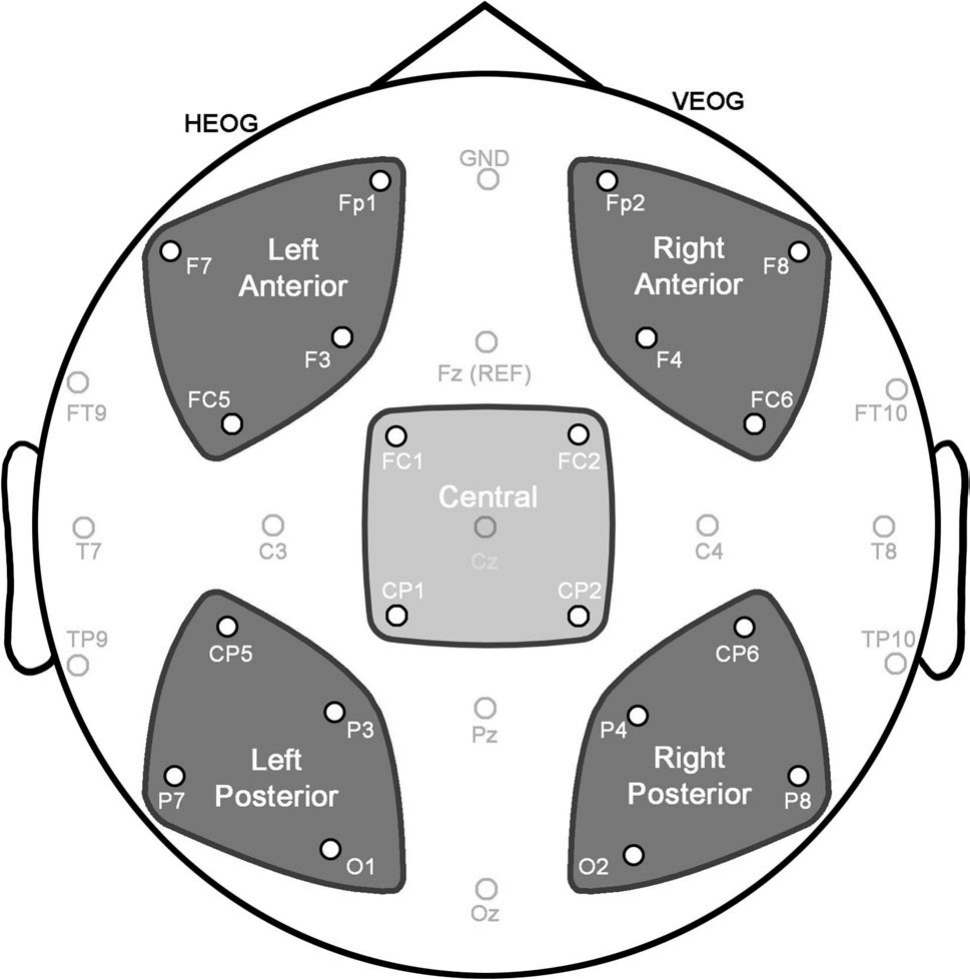
Central and peripheral regions of interest used in the analysis, with each region averaging across four electrodes. Peripheral regions were compared across factors of Hemisphere and Anterior-Posterior Distribution

Our ERP analysis focused on measurements recorded to the final face–upfix dyad. We analyzed the epochs of 200–300 ms, 300–500 ms, and 500–700 ms, corresponding to ERP components of the N250, N400, and later effects (sustained negativities, late positivities). We averaged across four electrode sites in each of five regions of interest with broad coverage of the scalp including a central region (FC1, FC2, CP1, CP2), and peripheral regions of the left anterior (Fp1, F7, F3, FC5), right anterior (Fp2, F8, F4, FC6), left posterior (CP5, P3, P7, O1), and right anterior (CP6, P4, P8, O2), as depicted in Fig. 5. For each epoch, we used repeated-measures ANOVAs with factors of Conventionality (2: conventional, unconventional) and Matching (2: match, mismatch), and for peripheral regions divided the scalp into factors of Hemisphere (2: left, right) and Anterior-Posterior (AP) Distribution (2: anterior, posterior). These electrode sites are depicted in Fig. 5. Significant interactions in the analysis of peripheral regions were followed by ANOVAs comparing Conventionality and Matching within each peripheral region, with post hoc comparisons using a Bonferroni correction.

As in Experiment 1, a follow up analysis correlated the brain response for both matching and mismatching dyads for each epoch with prior ratings of conventionality. Here, we calculated the ERP response to each item for each participant, and then averaged across participants for each dyad type for the electrodes used in regions of interest for each epoch (200–300, 300–500, 500–700). We then used Pearson’s correlations set to an alpha of .05 to compare the conventionality scores from our previous study (as in Experiment 1) with these averaged ERP responses for matching and mismatching dyads. Additional correlations with VLFI scores examined the interaction of these findings with comic reading expertise.

### Results

#### Behavioral results

**Fig. 6:**
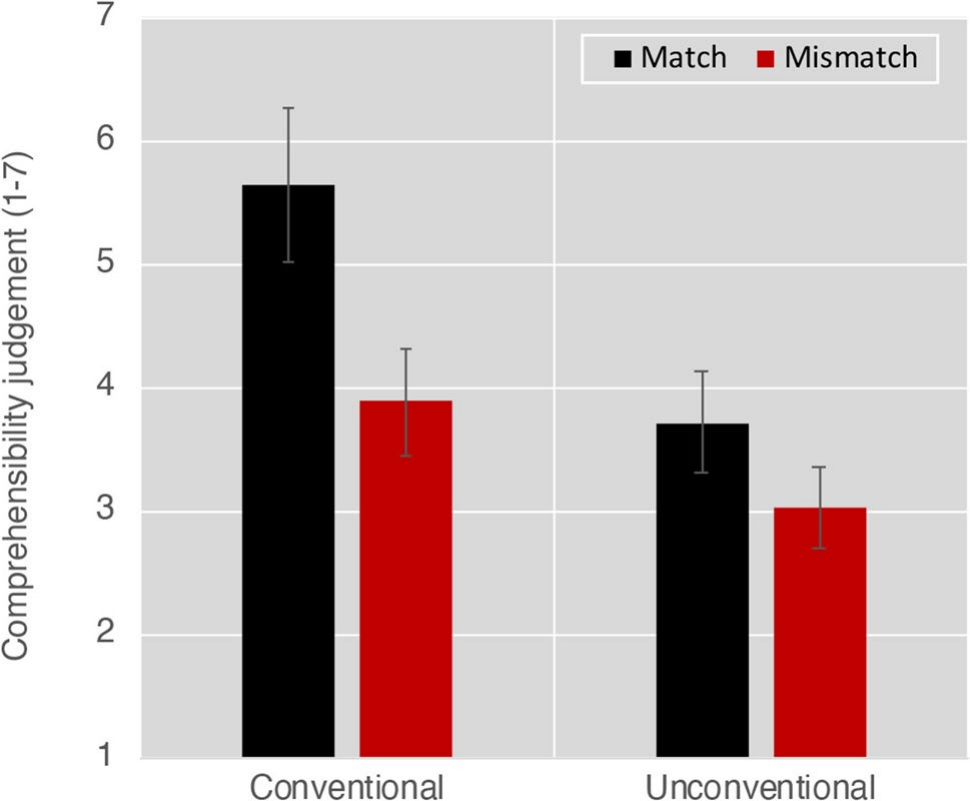
Results of comprehensibility ratings (1 = *incomprehensible*, 7 = *comprehensible*) to assessments of face–upfix dyads that varied on dimensions of conventionality and the matching of the facial expression to the upfix. Error bars depict standard error

Analysis of comprehensibility ratings for upfixes showed main effects of Conventionality and Matching, and an interaction between them (all *F*s > 49.3, all *p*s < .001). This arose because conventional matching dyads were rated as the most comprehensible, unconventional mismatching dyads as the least comprehensible, and unconventional matching and conventional mismatching dyads as in-between, as depicted in Fig. 6. All contrasts were significantly different from each other (all *p*s < .001), except for the intermediate ratings of unconventional matching dyads and conventional mismatching dyads. This is highly consistent with the ratings in Experiment 1.

An additional positive correlation arose between VLFI scores and the difference between ratings of conventionality (unconventional minus conventional), *r*(18) = .572, *p* < .01. This indicated that participants with greater comic reading experience had a bigger difference in their ratings between conventional and unconventional dyads.

In posttest questionnaires asking whether they noticed anything “unusual” about the stimuli, 38% of participants noticed mismatching dyads, overtly stating that “some pairs did not make sense,” “some signs did not match the face,” and “some of the combinations seemed wrong.” In addition, 50% mentioned aspects of unconventionality, such as that certain affixes were “weird” (like the fish).

#### EEG results

**Fig. 7:**
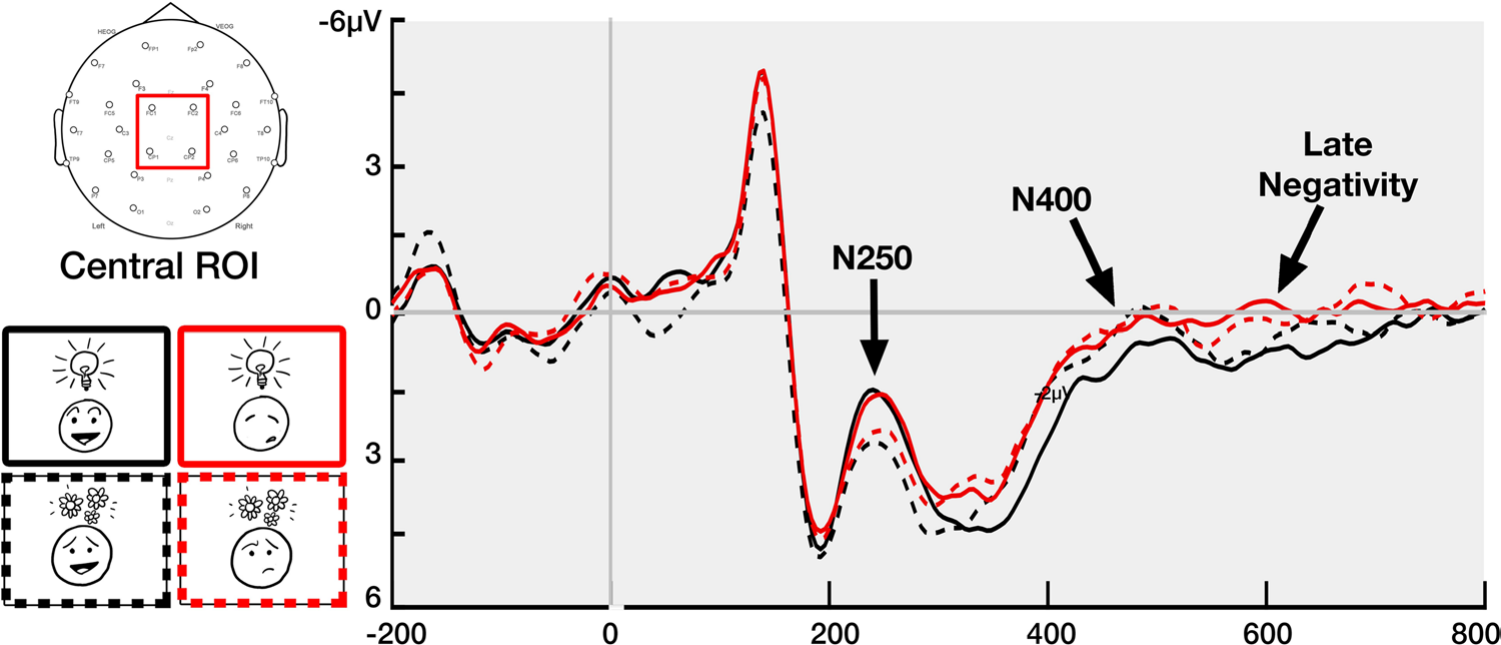
Grand average ERPs across participants for face–upfix dyads varying conventionality (conventional vs. unconventional) and face–upfix matching (match vs. mismatch) averaged across the 4 electrodes of the central region of interest, and topographic voltage maps showing the scalp distribution of the differential effects between conventionality and matching

**Fig. 8:**
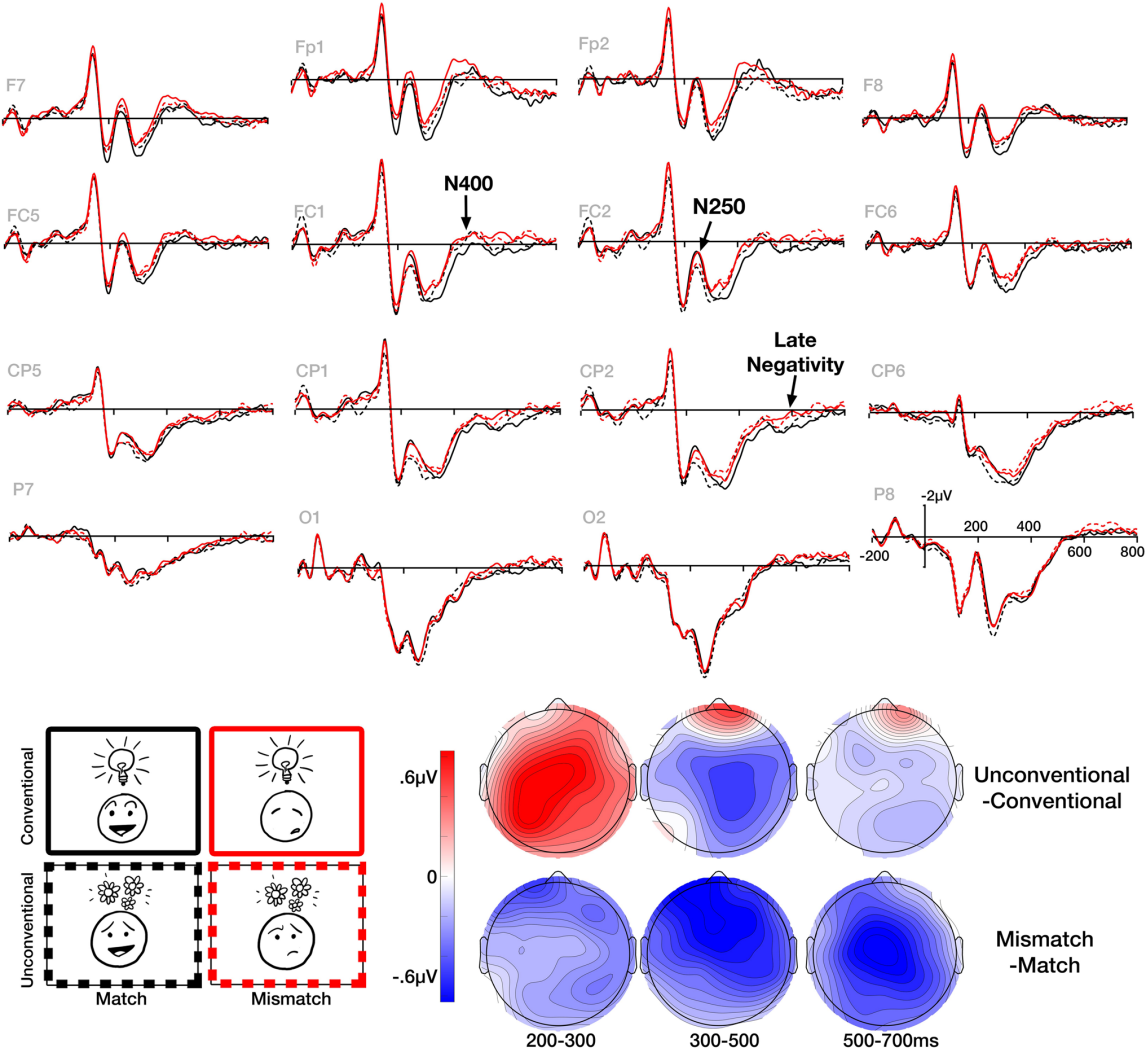
Grand average ERPs across participants for face–upfix dyads varying conventionality (conventional vs. unconventional) and face–upfix matching (match vs. mismatch) across 16 electrodes of the scalp, and topographic voltage maps showing the scalp distribution of the differential effects between conventionality and matching

Results of all statistical analyses are provided in Table 2. Figure 7 depicts EEG averaged across electrodes in the central region, while Fig. 8 depicts the 16 electrodes averaged in our analysis along with topographic maps.

**Table 2:** Results of ANOVAs comparing face–upfix dyads across Matching (M) and Conventionality (C) for both central and peripheral regions of interest

	200-300ms				300-500ms				500-700ms			
	Central		Peripheral		Central		Peripheral		Central		Peripheral	
	F	η^2^_p_	F	η^2^_p_	F	η^2^_p_	F	η^2^_p_	F	η^2^_p_	F	η^2^_p_
M	8.8**	0.32	3.8^	0.17	9.3**	0.13	9.6**	0.34	12.1**	0.15	3.8^	0.17
C	17.3***	0.48	6.8*	0.27	4.7*	0.07	0.59	0.03	0.27	0.01	0.11	0.01
M*C	10.2**	0.35	0.18	0.01	1.7	0.02	1.8	0.09	1.2	0.01	0.35	0.02
M*AP			0.93	0.05			1.8	0.09			0.09	0.004
C*AP			0.86	0.04			0.25	0.01			0.43	0.02
M*H			0.09	0.01			0.002	<.001			0.26	0.01
C*H			0.12	0.01			1.4	0.07			0.01	<.001
M*C*AP			4.2^	0.18			1.8	0.09			0.01	<.001
M*C*H			5.4*	0.22			0.62	0.03			2.8	0.13
M*AP*H			1.5	0.07			3.5^	0.16			6.3*	0.25
C*AP*H			6.1*	0.2			2.1^	0.14			1.99	0.1
M*C*AP*H			1.2	0.06			0.3	0.02			4.0^	0.17

*H* Hemisphere, *AP* Anterior-Posterior Distribution. ^*p* < .1, *p < .05, **p < .01, ***p < .001. *df* = 1, 19

In the 200–300-ms epoch, we observed main effects of Conventionality in both central and peripheral regions and a Conventionality × AP × Hemisphere interaction in peripheral regions. This arose because the conventional mismatching dyads maintained a similar amplitude to conventional matching dyads, but both evoked a greater negativity than the unconventional dyads. We further observed a main effect of Matching in the central region, and an interaction of Matching × Conventionality × Hemisphere in peripheral regions. These interactions were followed by ANOVAs in each region which found a main effect of Conventionality in both posterior regions (all *F*s > 8, all *p*s < .05, all η_p_^2^s > .29) and a Matching × Conventionality interaction in the right posterior region, *F*(1, 19) = 9.02, *p* < .01, η_p_^2^ = .322. These interactions clarified that, overall, the conventional matching and mismatching dyads evoked greater negativities than both unconventional dyads. However, in the left posterior region, unconventional matching dyads were slightly more attenuated than unconventional mismatching dyads (*t* = 2.9, *p* < .05).

Analyses in the 300–500-ms epoch revealed main effects of Matching and Conventionality in the central region, and a main effect of Matching in the peripheral regions. These effects arose because both mismatching dyads and unconventional matching dyads evoked a larger a fronto-central negativity (N400) than the conventional matching dyads. Unconventional matching dyads differed from both conventional and unconventional mismatches only in the distribution of the effect: mismatching dyads of both types had a larger and more widely distributed N400 than unconventional matching dyads. Mismatching dyads did not differ between conventional and unconventional dyads.

Finally, in the 500–700-ms epoch we observed a main effect of Matching in the central region. In the peripheral regions, a main effect of Matching came close but did not exceed the threshold of significance in peripheral regions (*p* = .066). A Matching × AP Distribution × Hemisphere interaction was followed by targeted ANOVAs in each region, revealing significant main effects of Matching in both posterior regions (all *F*s > 6.2, all *p*s < .05, all η_p_^2^s > .24), but no effects or interactions related Conventionality. These results arose because mismatching dyads evoked a larger sustaining central-parietal negativity than matching dyads, but this effect did not differ between conventional and unconventional dyads. This meant that unconventional matching dyads maintained the same amplitude relative to the conventional matching dads, but both conventional and unconventional mismatching dyads had a larger negativity.

**Fig. 9:**
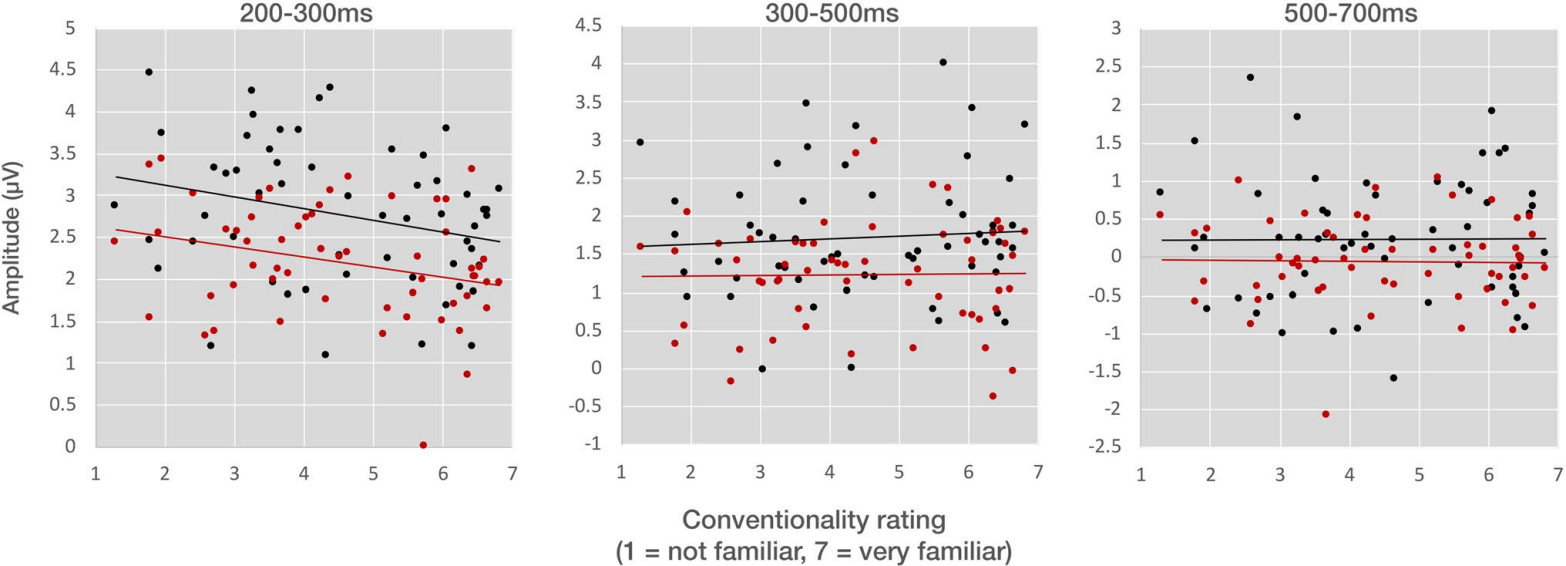
ERP amplitudes averaged across participants for each face–upfix dyad, correlated with conventionality ratings from Cohn et al. (2016) in the 200–300, 300–500, and 500–700 ms epochs for matching (black) and mismatching (red) face–upfix dyads. Each datapoint indicates a different dyad. *Note.* Amplitude scales differ for each epoch.

We then calculated the average ERP amplitude for each item across participants and correlated the mean amplitudes in each epoch with the conventionality scores. Significant negative correlations were found between conventionality scores and amplitudes in the 200–300-ms epoch, within both matching, r(56) = −.270, *p* < .05, and mismatching face–upfix dyads, *r*(56) = −2.79, *p* < .05. No correlations were found in the 300–500 or 500–700-ms epochs (all *p*s > .63). As in Fig. 9, these correlations in the 200–300-ms epoch suggested that more conventional dyads evoked more negative amplitude ERPs.

### Discussion

This experiment measured ERPs to visual morphology to investigate the neurocognition of combinatorial processing in visual affixes involving faces, as found in comics and emoji. Differences between dyads first emerged with a larger N250 to conventional dyads than unconventional ones. This effect correlated with ratings of the conventionality of upfixes. A subsequent N400 was then greater to unconventional and mismatching face–upfix dyads than to conventional dyads, and finally a sustained negativity was greater to mismatching than matching face–upfix relationships. These results suggest cascading processes that differentially respond to the manipulations of conventionality and matching in face–upfix relationships.

In the early, 200–300-ms epoch we found a greater leftward posterior negativity to conventional face–upfix dyads than to unconventional dyads, regardless of the matching between face and upfix. This negativity was further clarified by correlating these amplitudes with conventionality scores from Cohn et al. (2016). Here, a graded response indicated that greater negativities were evoked by more conventional dyads. This greater negativity to conventional upfixes is similar to the enhanced negativity observed in Kendall (2019) after participants underwent training to associate symbols replacing a mouth with combinatorial meanings. This enhanced negativity in this prior study could thus be interpreted as demonstrating how the learning process turns an unconventional visual morpheme into a conventional one, consistent with studies of categorization and familiarity in visual object processing (Folstein et al., 2017; Scott et al., 2006).

Our observed negativity to conventionality lasted from 200 to 300ms with a phasic peak around 250 ms, consistent with the N250 (Begleiter et al., 1995; Schweinberger et al., 1995; Tanaka et al., 2006). While the N250 was first associated with familiarity to faces, it has been further interpreted as related to the access or formation of categories in visual representations (Folstein et al., 2017; Schendan & Ganis, 2015), particularly implicating a role of perceptual expertise (Jones et al., 2018). Consistent with that interpretation, conventionality scores modulated our observed N250 in a graded way. Here, we did not train participants on the meanings of visual morphemes (Folstein et al., 2017; Kendall, 2019), but rather the N250 was modulated by an assessment of participant familiarity with face–upfix dyads (Cohn et al., 2016); that is, a measure of entrenchment of these items in memory. Given the association of the N250 with face processing, it is worth noting that, while faces are involved in these dyads, the conventionality ratings are primarily motivated by the *upfixes*, not the faces, particularly given that matching had little effect on the observed N250.

In the subsequent time window between 300 and 500 ms, we observed greater N400s to unconventional and mismatching face–upfix dyads than conventional matching dyads. This negativity is consistent with prior findings of N400s to mismatching object relationships or incongruous objects in scenes (Barrett & Rugg, 1990; McPherson & Holcomb, 1999; Võ & Wolfe, 2013). Such effects here to unconventional and mismatching dyads indeed imply that they evoked greater costs to semantic processing than conventional dyads, though this did not correlate with conventionality scores, like the N250. In addition, no differences arose between the N400 effects to unconventional and mismatching dyads, nor was an additive effect observed to the combination of these factors in unconventional mismatches. This implies that any deviation from the entrenched, conventional face–upfix relationships incurs comparable costs to accessing or retrieving the meaning of the representations.

Finally, in the later 500–700-ms epoch we observed sustained negativities only to mismatching compared with matching dyads but which did not differ between conventional and unconventional dyads. Sustained negativities have been observed following N400s to incongruities introduced into visual narratives (West & Holcomb, 2002). These sustained negativities have thus far been associated with prolonged interpretive or inferential processing in both sentences (Baggio, 2018; Bott, 2010), compound words (Fiorentino et al., 2014), and visual narrative sequences (Cohn, 2020b). This neurocognitive evidence therefore suggests that mismatches introduce violations into face–upfix relationships with costs that persist beyond the access of semantic information in the N400. Indeed, despite their larger N400s, unconventional matching dyads attenuated to be similar to conventional upfixes in this later epoch. Because there was no modulation of this mismatching effect by conventionality, it implies that unconventional matching dyads maintained a coherency that was not found in the mismatching unconventional dyads.

Interestingly, in this later time window, mismatching or unconventionality did not evoke a late posterior positivity like the P600, which often characterizes morphosyntactic violations in language (Leminen et al., 2019). We do not take this to be a modality difference, as visual morphological violations of motion lines in the context of narrative sequences have evoked such late parietal positivities (Cohn & Maher, 2015). While such decontextualized presentation may have had an influence, our behavioral findings at least support participants do find mismatches as incongruous. One possibility is that, though the graphic arrangement of morphemes was not known in our study prior to the dyad (i.e., whether the affix would go above the head or in another place), its component parts were known prior to this critical position. Thus, participants would have been exposed to the mismatching elements before seeing the full dyad, which may have ameliorated updating or revision processes thought to be reflected by posterior positivities (Baggio, 2018; Cohn, 2020b; Donchin & Coles, 1988; Kuperberg, 2016).

These results provide additional support for the productive characteristics of upfixes as using a schematic template. Unconventional upfixes did incur a cost for being novel (N400) but are processed comparably to conventional upfixes once this initial unfamiliarity is resolved and their structural properties are recognized as comprehensible. In contrast, if the upfixes mismatch with the face—no matter their conventionality—they incurred sustained processing costs. Again, because this processing appears to sustain for violations of both conventional and unconventional upfixes, and not for matching ones of either conventionality, it further supports the productiveness of a broader schema for these dyads.

## General discussion

We examined the (neuro)cognition of combinatorial visual morphology in the form of face–upfix relationships across two studies examining behavioral reaction times and event-related brain potentials. While these experiments showed evidence for entrenched knowledge stored in memory (full-entry theory) and for computation of novel meanings (dynamic-construal theory), altogether these findings support that face–upfix dyads involve a schematic productive class, consistent with Visual Language Theory (Cohn, 2013, 2018).

A persistent finding in our experiments was the advantage of conventional dyads compared with all other types. In line with the expectations of a full-entry theory (Feng & O’Halloran, 2012; Kennedy, 1982; McCloud, 1993; Ojha, 2013; Walker, 1980), the findings that conventional dyads remain comprehended better in all contexts shows that they are stored in memory. In addition, the correlations with conventionality scores further emphasize that the degree of entrenchment matters for processing. However, the costs to mismatching face–upfix relationships suggest against a strong full-entry theory, since faces should be relatively less important if the upfix alone motivates the stored meaning.

At the same time, people can derive new meanings for upfixes in novel cases, as would be the expectation of a dynamic-construal theory (Bateman & Wildfeuer, 2014). Indeed, unconventional matching dyads were responded to faster (Experiment 1) and rated as more comprehensible (Experiments 1 and 2) than mismatching dyads, suggesting that unconventional upfixes can allow comprehensibility so long as they remain congruent with their corresponding faces. Yet, such construal does not seem to be uniform, as would be expected if comprehending all face–upfix relationships involved construing “defeasible interpretations.” Rather, we observed clear differences across the conventionality of matching dyads in both experiments, with further costs to mismatching face–upfix relationships.

In contrast to these two extremes of full storage versus full construal, our finding supports the idea that upfixes have become abstracted into a productive schema, which then manifests in various conventionalized forms (Cohn, 2013, 2018). Consistent with prior findings (Cohn et al., 2016), ratings of face–upfix dyads in both studies showed an interaction pattern where conventional upfixes were rated as more comprehensible than unconventional ones, but matches were more comprehensible than mismatches. Because mismatching dyads were rated as less comprehensible than matching dyads, but were also modulated by conventionality, it suggests that such constraints operate on novel upfixes as well as entrenched ones, as would be expected of an abstracted pattern.

Nevertheless, in Experiment 2 the pattern of ERP amplitudes differed from both response times and ratings. Rather than a uniform difference between amplitudes of matching and conventionality conditions across the time course, modulations occurred in specific components which have been linked to understanding visual and linguistic information. An early difference arose between a larger negativity to conventional than unconventional dyads. As this negativity was indicative of the N250, it suggests “perceptual expertise” for accessing the conventional representations (Folstein et al., 2017; Schendan & Ganis, 2015). This gave way to subsequent differences in the N400, where both unconventional and mismatching upfix dyads evoked greater N400s than the conventional matching dyads. Given that the N400 is an index of semantic processing (Kutas & Federmeier, 2011), it implies that such access or retrieval of meaning is not differentially nor additively affected by mismatching or conventionality—they had a seemingly equivalent effect. Rather, mismatching further elicited a later negativity, which here was indeed evoked regardless of conventionality, perhaps reflecting a process of sustained interpretation (Baggio, 2018; Bott, 2010; West & Holcomb, 2002). Thus, the response times and ratings in Experiment 1 were likely motivated by a mix of the processes across neural responses.

Taken together, these results across the time course of processing further support a schematic interpretation. At the N400, both unconventionality and matching factor into accessing semantic representations, where conventional matching dyads attenuate processing because of their encoding in memory. This suggests that the combinatorial meaning of face–upfix relationships is immediately apparent and accessible, given that mismatching evoked larger N400s than conventional matching dyads. Yet, with these accessed semantic representations, mismatching face–upfix relationships sustained into later negativities compared with matching dyads. This finding suggests that, despite the greater cost of semantic processing (N400), unconventional matches are resolvable, but mismatches are not, no matter their conventionality. As this later negativity indicates that novel forms can be construed yet are still subject to costs when violated with mismatches, it supports our proposal of a productive, yet constrained, abstract schema.

This schema theory of face–upfix dyads allows for novel forms to productively be interpreted, but it also affords the encoding of stored forms that fill (and possibly motivate) the schema. Such forms may thus be encoded in memory with a range of familiarity, as evident in our graded conventionality ratings from our prior study (Cohn et al., 2016), which in turn correlated with measurements in both experiments to further highlight how familiarity enhances processing. We first observed this in Experiment 1 with graded responses appearing to the response times only for matching dyads, but not mismatches. This implied there was no benefit of conventionality to judging the comprehensibility of face–upfix dyads when their relationship appears violated. An additional correlation with conventionality ratings appeared to the amplitudes of the N250 for both matching and mismatching dyads, but not with amplitudes in later time windows. This suggested that the faster response times in Experiment 1 to matches alone were motivated by this fairly early component, while later neural processes affecting mismatching (N400, sustained negativities) may have obviated the graded response times to mismatching dyads.

These findings for graded behavioral and neural responses provide evidence of the entrenchment of these visual lexical items in memory in a full-entry way, no matter the relationship between face and upfix (Feng & O’Halloran, 2012; Forceville, 2011, 2016; Kennedy, 1982; McCloud, 1993; Ojha, 2013; Walker, 1980). Furthermore, the early time window of this N250 response suggests that this encoding primarily relates to expertise with the percepts themselves (Folstein et al., 2017; Schendan & Ganis, 2015), while the subsequent semantic processing (N400) is not as sensitive to conventionality in such a graded way. Rather, later negativities indexing more stored semantic (N400) or interpretive processes (sustained negativities) were greater for unconventional and/or mismatching dyads, but with no graded variation with conventionality scores. The insensitivity to conventionality scores in the later negativities and their sensitivity to mismatching suggests upfixes alone do not motivate understanding of the dyad. Rather, interpretation involves both conventionality of the upfix and its relationship to the facial expression.

Finally, modulation of our findings based on participants’ expertise further supports the role of familiarity and entrenchment of upfix meanings. We observed a greater difference between ratings of conventional and unconventional dyads for participants with higher VLFI scores in Experiment 2. These findings are consistent with our prior findings of ratings for face–upfix dyads and comics reading expertise (Cohn et al., 2016), and with earlier work finding that understanding of upfixes was modulated by the frequency with which they appear in comics (Newton, 1985).

In sum, these findings lend support to combinatorial visual morphemes involving a combination of information stored in memory and productive creation of novel forms. Such a structure aligns with contemporary views of linguistic competence, and with arguments that graphic information also involves a lexicon comparable to that of language.
